# Decreased influenza-specific B cell responses in rheumatoid arthritis patients treated with anti-tumor necrosis factor

**DOI:** 10.1186/ar3542

**Published:** 2011-12-16

**Authors:** James J Kobie, Bo Zheng, Peter Bryk, Michael Barnes, Christopher T Ritchlin, Darren A Tabechian, Allen P Anandarajah, R John Looney, Ralf G Thiele, Jennifer H Anolik, Andreea Coca, Chungwen Wei, Alexander F Rosenberg, Changyong Feng, John J Treanor, F Eun-Hyung Lee, Ignacio Sanz

**Affiliations:** 1 Division of Allergy, Immunology and Rheumatology, University of Rochester Medical Center, 601 Elmwood Avenue, Box 695, Rochester, NY, 14642, USA; 2 Division of Pulmonary & Critical Care Medicine, University of Rochester Medical Center, 601 Elmwood Avenue, Box 692, Rochester, NY, 14642, USA; 3 Department of Biostatistics and Computational Biology, University of Rochester Medical Center, 601 Elmwood Avenue, Box 630, Rochester, NY, 14642, USA; 4 Division of Infectious Disease, University of Rochester Medical Center, 601 Elmwood Avenue, Box 689, Rochester, NY, 14642, USA

## Abstract

**Introduction:**

As a group, rheumatoid arthritis (RA) patients exhibit increased risk of infection, and those treated with anti-tumor necrosis factor (TNF) therapy are at further risk. This increased susceptibility may result from a compromised humoral immune response. Therefore, we asked if short-term effector (d5-d10) and memory (1 month or later) B cell responses to antigen were compromised in RA patients treated with anti-TNF therapy.

**Methods:**

Peripheral blood samples were obtained from RA patients, including a subset treated with anti-TNF, and from healthy controls to examine influenza-specific responses following seasonal influenza vaccination. Serum antibody was measured by hemagglutination inhibition assay. The frequency of influenza vaccine-specific antibody secreting cells and memory B cells was measured by EliSpot. Plasmablast (CD19+IgD-CD27hiCD38hi) induction was measured by flow cytometry.

**Results:**

Compared with healthy controls, RA patients treated with anti-TNF exhibited significantly decreased influenza-specific serum antibody and memory B cell responses throughout multiple years of the study. The short-term influenza-specific effector B cell response was also significantly decreased in RA patients treated with anti-TNF as compared with healthy controls, and correlated with decreased influenza-specific memory B cells and serum antibody present at one month following vaccination.

**Conclusions:**

RA patients treated with anti-TNF exhibit a compromised immune response to influenza vaccine, consisting of impaired effector and consequently memory B cell and antibody responses. The results suggest that the increased incidence and severity of infection observed in this patient population could be a consequence of diminished antigen-responsiveness. Therefore, this patient population would likely benefit from repeat vaccination and from vaccines with enhanced immunogenicity.

## Introduction

TNFα is a potent pro-inflammatory cytokine produced by macrophages, T, B, and dendritic cells, having pleiotropic effects on the immune system, including the development and progression of autoimmune diseases. TNF blockade has been extremely effective in treating multiple inflammatory diseases, including rheumatoid arthritis (RA); however, chronic blockade of TNF may increase the risk of infections [1,2], including bacterial pathogens such as tuberculosis, fungal infections, and viral infections including herpes zoster and human papillomavirus [2,3]. Furthermore, several studies have reported reduced induction of serum antibodies in patients treated with anti-TNF following vaccination against influenza virus and pneumococcal bacteria [4-6]. Methotrexate (MTX), which inhibits folate metabolism and promotes the production of immunosuppressive extracellular adenosine, is commonly used to treat RA [7]. Thus, anti-TNF treatment either alone or in combination with MTX may contribute to reduced immune responses to infections and vaccination by limiting B cell responses and subsequent development of protective serum antibodies.

TNF impacts B-cell repertoire development and homeostasis, as well as B cell responsiveness by multiple direct and indirect mechanisms. This effect includes direct modulation of B cell activation and survival through nuclear factor (NF) κB activation after the cytokine binds surface TNFRI and TNFRII [8]. Surface-bound TNF on activated macrophages and monocytes can activate CD4+ T cells via TNFR and thus support T-dependent B cell responses. Additionally, it has been demonstrated that TNF mobilizes mouse bone marrow B cells to the blood and spleen by suppressing stromal CXCL12 retention signals in the bone marrow [9,10]. During an inflammatory response this could promote bone marrow granulopoiesis and extramedullary lymphopoiesis, the former of which most likely plays a critical role in control of infection. Importantly, TNF has a key role in follicular dendritic cell organization and function, and in germinal center reactions [11,12], and we have previously demonstrated that TNF blockade with etanercept in RA patients profoundly diminishes the follicular dendritic cell network and disrupts germinal center reactions [13]. Because germinal center reactions are critical for optimal antibody induction, we postulate that TNF blockade alters the effector and memory B cell responses, contributing to increased risk of infection and poor response to vaccination.

Following vaccination there is a transient effector B cell response including the expansion of B cell plasmablasts, defined as CD19+IgD-CD27hiCD38hi cells that can be readily observed in the peripheral blood and strongly correlate with the vaccine-specific antibody-secreting cell response [14-16]. In response to a recall antigen such as influenza vaccine, the effector peak is typically between five to seven days and slightly later in a primary response, with a return to the steady state within 14 days after immunization. This transient plasmablast population is highly enriched for B cells actively secreting antibody against the immunogen [14,17] and may contain precursors to the long-lived CD138+ mature plasma cells that reside in the bone marrow and are the presumptive source of serum antibodies present months and years following vaccination [15,18]. Additionally, following vaccination, antigen-specific memory B cells develop, persist, and circulate throughout the periphery, readied to differentiate into antibody-secreting plasmablasts and plasma cells upon re-exposure to antigen.

Anti-TNF has minimal effect on the ability of RA patients to achieve the standard 40 or higher protective titer after influenza vaccination [4,5,19,20]; however, lower geometric mean titers (GMT) of antibody have been observed [4-6]. These observations suggest that treatment of RA patients with anti-TNF can result in sub-optimal vaccine responses. In this study we specifically examined the impact of anti-TNF treatment of RA patients on influenza-specific B cell responses. We demonstrate that the majority of RA patients treated with anti-TNF have a decreased influenza-specific effector B cell response that correlates with diminished development of memory B cells and serum antibodies.

## Materials and methods

### Patients

We enrolled 261 subjects, including 164 patients with diagnosed RA and 97 healthy control subjects at the University of Rochester Medical Center from 2006 to 2010 who were all receiving seasonal inactivated trivalent influenza vaccine (TIV) as standard-of-care. Patients provided signed written informed consent. All procedures and methods were approved by the Research Subjects Review Board at the University of Rochester Medical Center.

### Sampling

Peripheral blood was obtained from subjects at one time point prior to receiving TIV, and then at one and six months post-vaccination. This cycle was repeated for two seasons for each subject. Based on subject willingness, availability, and logistical constraints, a subset of subjects provided two additional samples collected following 2009-2010 TIV immunization; one obtained on day five to day seven post-vaccination, and another obtained day eight to day ten post-vaccination. A limited number of samples were also obtained from subjects day five to day seven following 2010-2011 TIV immunization.

PBMC were isolated within two hours of sampling using CPT tubes (Becton Dickinson, Franklin Lakes, NJ, USA). Tubes were immediately inverted 8 to 10 times and processed according to manufacturer's instructions. Peripheral blood mononuclear cells (PBMCs) were cryopreserved and stored in liquid nitrogen. Serum was collected, aliquotted and stored at -80°C. All sample processing was performed in a blinded manner.

### Hemagglutination inhibition assay

A standard hemagglutination inhibition assay was performed in a blinded manner as previously described [21]. Briefly, serial two-fold serum dilutions were assayed against influenza H1N1, H3N2, and B using strains contained in corresponding year's TIV vaccine or appropriately matched closely related strain.

### Memory B cell culture

Total B cells were isolated from fresh PBMC by negative magnetic bead selection of non-B cells according to manufacturer's protocol (Miltenyi Biotec, Auburn, CA, USA) and cryopreserved. B cell samples were thawed, washed once in culture media (RPMI containing 10% heat-inactivated fetal bovine serum and antibiotic/antimycotic), and 30,000 cells added per well of a 96-well round-bottom plate in 200 μl culture media containing 2.5 μg/ml CpG ODN 2006 (Oligos Etc, Wilsonville, OR, USA) and 10 ng/ml IL-2 (Peprotech, Rocky Hill, NJ, USA). Cells were cultured for four days at 37°C and 5% CO2.

### Antibody-secreting cell Elispots

The frequency of influenza antigen-specific antibody-secreting cells (ASCs) was measured by Elispot as previously described [16,22]. Briefly, 96-well Elispot plates (MAIPS4510 96 well, Millipore, Bedford, MA, USA) were coated overnight at 4°C in a humidified chamber with either: TIV Influenza Virus Vaccine (6 μg/mL, Sanofi Pasteur Inc., Swiftwater, PA, USA), anti-human IgG (5 μg/mL, Jackson Immunoresearch, West Grove, PA, USA), or anti-human IgM (5 μg/mL, Jackson Immunoresearch, West Grove, PA, USA). These antigens and capture antibodies were diluted in sterile PBS to above concentrations. Bovine serum albumin (2%) in sterile PBS was used as an irrelevant antigen. Coated plates were blocked with RPMI and 8% fetal bovine serum for two hours and incubated at 37°C for 18 to 20 hours with 300,000, 100,000, and 33,333 PBMC or with 25,000 and 2,500 cultured B cells per well in triplicate. For total-IgG and IgM plates, 100,000, 33,000, and 11,000 PBMC or 2,500 and 250 cultured B cells per well were added. After incubation, cells were aspirated and plates were washed with PBS with 0.1% Tween (PBST). Bound antibodies were detected with alkaline phosphatase-conjugated anti-human IgG or anti-human IgM antibody (1 μg/mL, Jackson Immunoresearch) for two hours and developed with VECTOR Blue, Alkaline Phosphatase Substrate Kit III (Vector Laboratories, Burlingame, CA, USA). Spots in each well were counted in a blinded manner using the CTL immunospot reader (Cellular Technologies Ltd, Shaker Heights, OH, USA).

### Flow cytometry

Cryopreserved peripheral blood lymphocytes were thawed and stained with anti-CD19-APC-Cy7 (SJ25C1, BD Biosciences, San Diego, CA, USA) anti-CD20-AlexaFluor 700 (2H7, Biolegend, San Diego, CA, USA), anti-CD3-PerCP-Cy5.5 (SP34-2, BD, anti-IgD-FITC (IA6-2, BDanti-IgM-PE-Cy5 (G20-127anti-CD27-Qdot605 (CLB-27/1, Invitrogen, Grand Island. NY, USA), anti-CD38-PE-Cy7 (HIT2, ebioscience, San Diego, CA, USA), anti-CD24-PE-AlexaFluor 610 (SN3, Invitrogenanti-CD1c-PE (AD5-8E7, Miltenyi Biotec, Auburn, CA, USA), anti-CD45R/B220-APC (RA3-6B2, BD), and Live/Dead fixable violet dead cell stain (Invitrogen). One-to-two million events per sample were collected on an LSRII instrument (BD Biosciences) and analysis performed in a blinded manner using FlowJo software (Treestar, Inc, Ashland, OR, USA). Total PBMC were gated on lymphocytes using FSC and SSC. To exclude dead cells, and T cells, Live/Dead stain, and anti-CD3 were used, respectively. Plasmablasts were identified within the CD19^+ ^B cell population as IgD-CD24-CD27++CD38++. Fresh lymphocytes were also stained in the same manner from samples obtained days five to seven after immunization with 2010-2011 TIV.

### Statistical analysis

Fisher's exact test was used to compare differences in the induction of serum antibodies and TIV-specific memory B cells among groups from multiple years. For multiple-year composite data, an individual subject may have provided data for two years. Kruskal-Wallis test was used to compare plasmablast, ASC, and serum antibody responses to the 2009-2010 TIV between groups. For correlative analysis of 2009-2010 HAI average change for each subject was determined as: ((H1 1mo/H1 base) + (H3 1mo/H3base) + (B 1mo/B base))/3. Spearman one-tailed correlation co-efficient was used to measure the correlation of two variables. Statistical analyses were performed using Prism 5.0 software (GraphPad Software, La Jolla, CA, USA) and SAS 9.2 (SAS Institute Inc., Cary, NC, USA).

## Results

### Study design and patient demographics

This multi-year study was designed to broadly assess the impact of anti-TNF therapy on B cell responses to influenza. Between 2006 and 2010 peripheral blood was obtained prior to (0), one, and six months following vaccination with seasonal trivalent inactivated influenza vaccine (TIV) administered as standard-of-care. For most subjects, this cycle was repeated for two seasons. As shown in Table 1, the RA+aTNF group (RA+aTNF) was comprised of patients receiving adalimumab (Humira), etanercept (Enbrel), or infliximab (Remicade) also as standard-of-care. Many of these subjects were concomitantly treated with MTX. The RA+MTX group consisted of patients treated with MTX, but not anti-TNF or any other biological therapy. There was no statistical difference in the MTX dose between the RA+aTNF and RA+MTX dose (not shown). The RA group (RA) consisted of patients not receiving MTX, anti-TNF, or any other biological therapy, and was predominantly comprised of recently diagnosed patients. A diverse group of healthy controls (HC) was enrolled encompassing a broad range of ages.

**Table 1:** Cohort demographics

	RA+aTNF	RA+MTX	RA	Healthy Controls
**N**	61	70	33	97
**Age, mean +/- SD years**	55.4 +/- 12.3	58.4 +/-12.2	57.1 +/-13.8	39.8 +/- 13.6
**% female**	82%	77%	64%	63%
**Disease duration < 1 year**	5%	17%	45%	NA
**Disease duration > 3 years**	93%	60%	47%	NA
**Adalimumab n (%)**	9 (15)	NA	NA	NA
**Infliximab n (%)**	17 (28)	NA	NA	NA
**Etanercept n (%)**	35 (57)	NA	NA	NA
**MTX n (%)**	49 (80)	70 (100)	0 (0)	NA
**MTX, dose +/- SD mg/wk**	15.2 +/- 4.3	16.5 +/- 4.0	NA	NA
**corticosteroid n (%)**	18 (29.5)	26 (37.1)	10 (30.3)	NA
**corticosteroid, dose +/- SD mg/d**	5.5 +/- 3.5	4.9 +/- 2.0	10.3 +/-6.1	NA
**ESR mean (range)**	21 (4-70)	22 (2-83)	20 (2-74)	NA
**HAQ mean (range)**	0.71 (0.00-2.22)	0.73 (0.00-2.75)	0.46 (0.00-1.63)	NA
**VAS mean (range) cm**	2.8 (0.0-9.8)	3.3 (0.0-7.5)	2.8 (0.0-7.1)	NA
**Morning stiffness mean (range) min**	81 (0-780)	104 (0-1440)	59 (0-180)	NA
**Race non Caucasian n (%)**	8 (13)	6 (9)	2 (6)	12 (12)

ESR, erythrocyte sedimentation rate; HAQ, health assessment questionnaire; MTX, methotrexate; NA, not applicable; SD, standard deviation; VAS, visual analogue score.

### Influenza-specific antibody and memory B cell response

**Figure 1:**
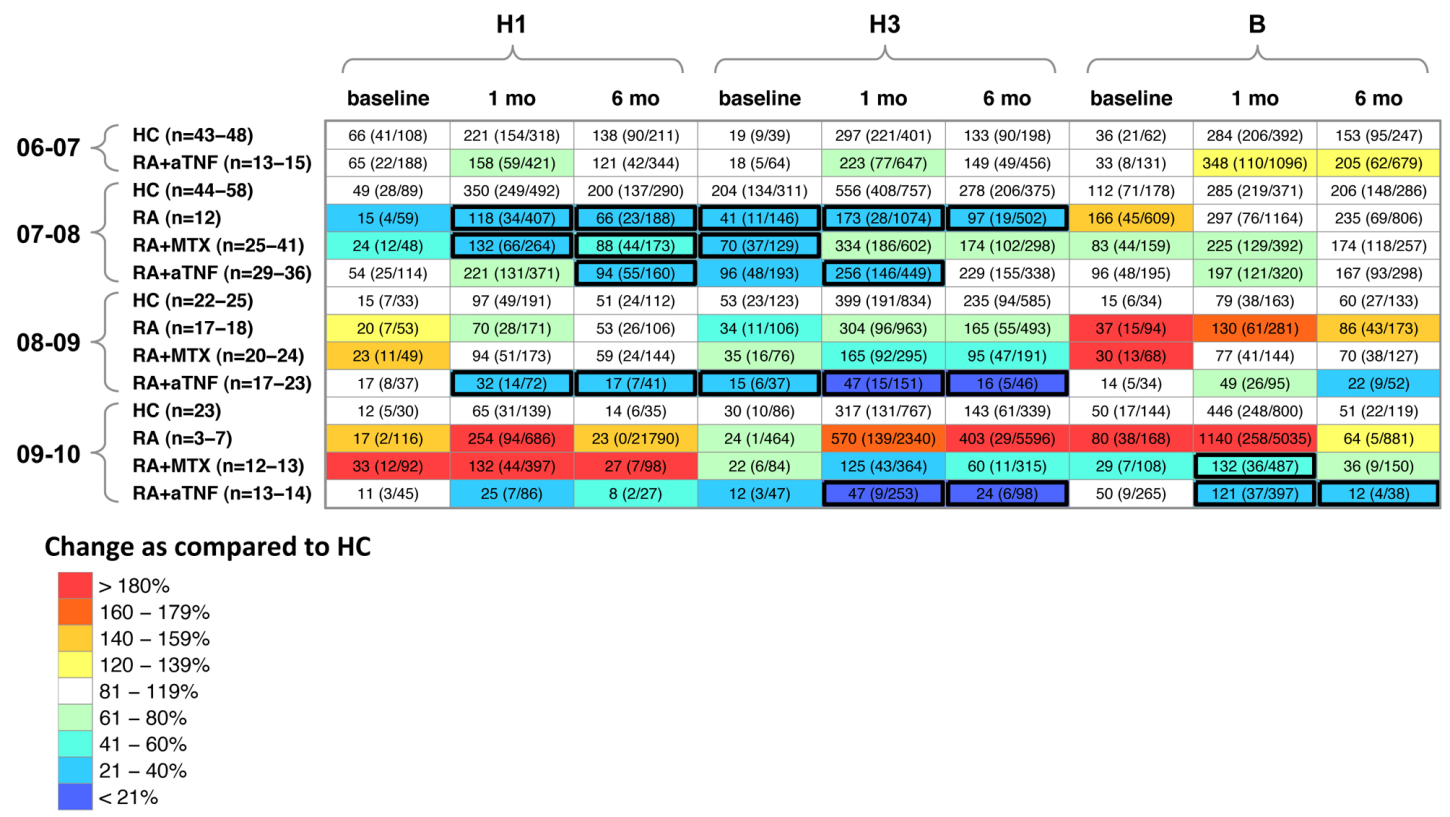
**Serum hemagglutination-inhibition antibody titers**. Peripheral blood was obtained prior to (baseline), and one and six months following vaccination with trivalent influenza vaccine (TIV) throughout multiple years. Influenza-specific serum antibody was measured by hemagglutination inhibition assay and geometric mean titer (GMT) determined for the indicated influenza subtype. GMT (upper 95% confidence interval (CI)/lower 95% CI) indicated. Relative difference in GMT among rheumatoid arthritis (RA) groups as compared with the healthy control (HC) group at same timepoint indicated by color. Cells with bold lines indicate significant difference (*P *< 0.05) compared with HC group at same timepoint. N, number of subjects measured per year. MTX, methotrexate.

To determine the serum antibody response to influenza vaccination the hemagglutination inhibition assay was performed. RA patients treated with anti-TNF had lower GMT of serum antibody compared with healthy controls in all study years (Figure 1). Specifically, at one month following vaccination, RA patients treated with anti-TNF had on average throughout all the study years 50%, 65%, and 30% lower H1, H3, and B GMT, respectively, compared with HC (Figure 1). At six months following vaccination, RA patients treated with anti-TNF had on average throughout all the study years 46%, 46%, and 31% lower H1, H3, and B GMT, respectively, compared with HC (Figure 1). Although lower GMT were observed in the RA+MTX group compared with the HC group, it was less consistent than that observed in the RA+aTNF group. Although variability throughout the study years was also observed in the post-vaccination GMT of the untreated RA group, interestingly, however, increased GMT were observed at several timepoints compared with the HC group. Notably, most subjects had serum titers that reached sero-protective levels at one month following vaccination as defined by HAI titer of 40 or higher [see Additional data file 1].

**Figure 2:**
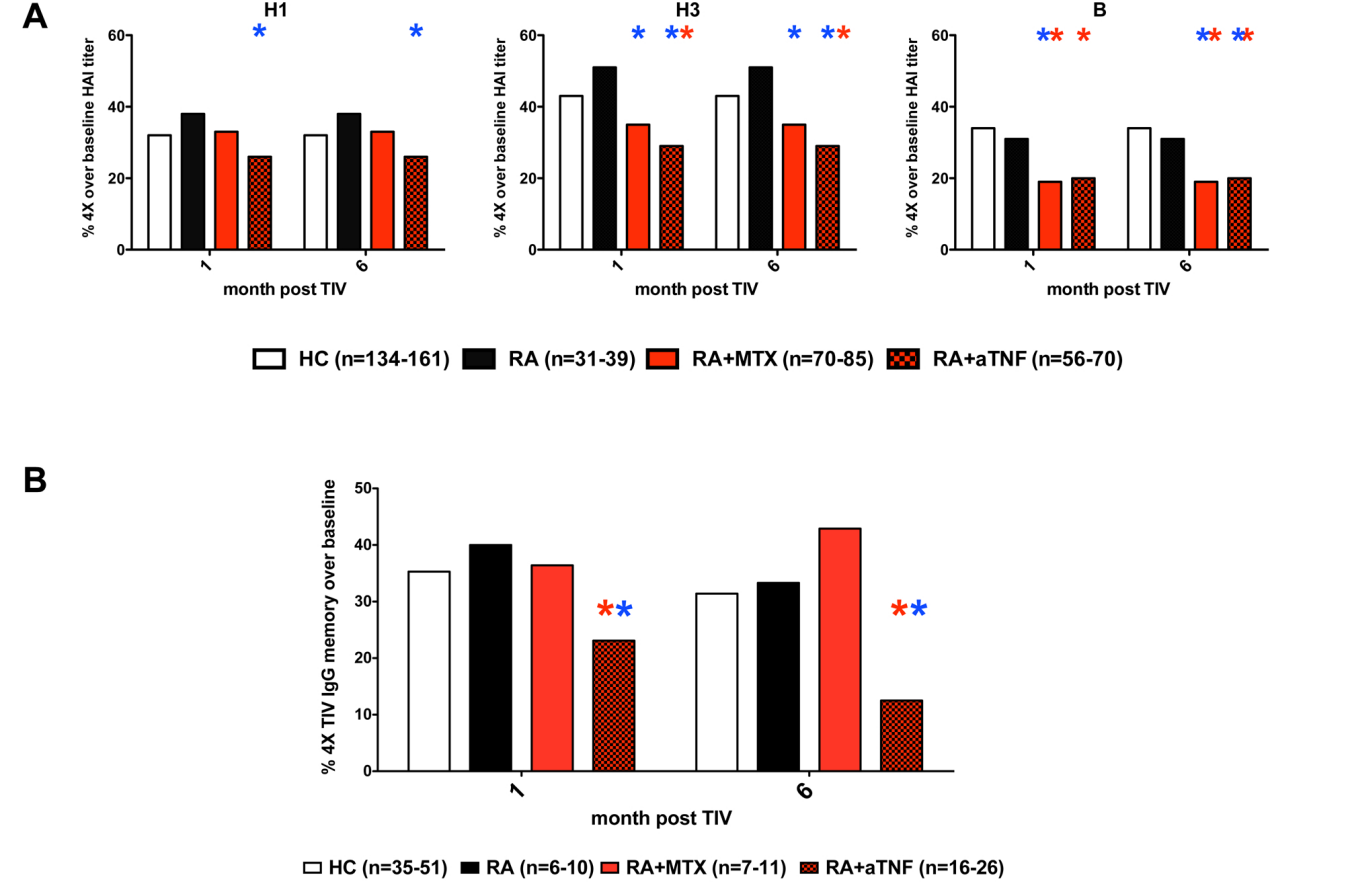
**Induction of serum antibodies and memory B cells**. (**a**) The frequency of subjects within each group that had a four-fold or greater increase in serum hemagglutination inhibition assay (HAI) titer over baseline was determined in 2006/2007 through 2009/2010 influenza seasons, and combined results indicated. (**b**) Total B cells were isolated, cultured with CpG and IL-2 for four days, and trivalent influenza vaccine (TIV) and total IgG specific EliSpots performed to determine the frequency of TIV-specific memory B cells. The frequency of subjects that had a four-fold or greater increase in the frequency of TIV-specific IgG memory B cells over baseline is presented. The red star indicates significant difference (*P *< 0.05) compared with healthy control (HC) group, the blue star indicates significant difference as compared with rheumatoid arthritis (RA) group. An individual subject may have provided data from multiple study years. N, the cumulative number of paired subject events measured throughout the multiple years.

We next determined the response rate to the influenza vaccine as defined by the frequency of subjects within each group that developed a four-fold or greater increase in HAI titer at one month or six months post vaccination over baseline. This analysis mitigated the impact of baseline GMT variation, which is likely to be due to differences in past influenza vaccination and natural infection among individuals, and facilitated group comparisons across multiple study years. A greater proportion of the untreated RA group responded to H1 and H3 as compared with the HC group at both one and six months post-vaccination (Figure 2a), and consistent with the annual results discussed above, this increase was sustained after six months in the untreated RA group. By contrast, fewer of the RA+aTNF patients responded to H1 at one and six months compared with the HC and RA groups. This difference only reached significance when the RA+anti-TNF group was compared with the untreated RA group. The RA+aTNF group and the RA+MTX group both had a lower proportion of patients responding to H3 compared with HC and RA. Both reached significance compared with RA, but only the RA+aTNF group was significantly lower than HC. Both the RA+MTX and RA+aTNF groups had a significantly lower proportion of patients that responded to influenza B compared with the HC and untreated RA groups. Therefore, the serum antibody response against multiple components of seasonal influenza vaccine is impaired in RA patients treated with anti-TNF. This effect may result from altered development, maintenance, or quality of influenza-specific plasma cells. As the influenza vaccine response is largely a secondary immune response, TNF blockade may be compromising the memory B cell response, perhaps by altering their maintenance or responsiveness.

To determine if anti-TNF therapy similarly affects the influenza-specific memory B cell response, TIV IgG EliSpots were performed from CpG and IL-2 stimulated B cells from a subset of patient samples. At baseline approximately 2.4% and 1.3% of the total IgG memory B cells for the HC and RA+aTNF groups respectively were TIV specific, but this difference was not significant (*P *= 0.097) [see Additional data file 2]. When examined as response following TIV, similar to the serum antibody response, the frequency of RA patients treated with anti-TNF that had a four-fold or greater increase in TIV-specific memory B cells at one month post-vaccination was significantly lower than both the HC and RA groups. Moreover, the influenza-specific memory B cell frequencies continued to decrease disproportionally at six months in anti-TNF treated patients as compared with all other groups. A modest increase was also observed in the frequency of untreated RA with a four-fold or greater increase in TIV-specific IgG memory B cells compared with the HC group (40% vs. 35% at one month, 33% vs. 31% at six months; Figure 2b). No significant difference was observed between the RA+MTX group and HC and RA groups. These results indicate that TNF blockade adversely impacts influenza-specific memory B cell responses to vaccination in RA patients both at the induction and maintenance phases of the memory response.

### Effector response to influenza vaccine

In HC, we have previously observed that the peak frequency of influenza-specific ASCs in the peripheral blood present shortly following vaccination correlates with the subsequent increase in serum hemagglutination inhibition (HAI) antibodies [16]. It is suggested that ASC are enriched in the peripheral blood CD19+IgD-CD27++CD38++ plasmablast population. We therefore asked if this effector response to influenza vaccination is also impacted by anti-TNF. In addition to baseline and one month samples, two peripheral blood samples were obtained from a subset of RA patients and healthy controls; one sample between day five and day seven, and a second between day eight and day ten following administration of the 2009-2010 seasonal influenza vaccine.

**Figure 3:**
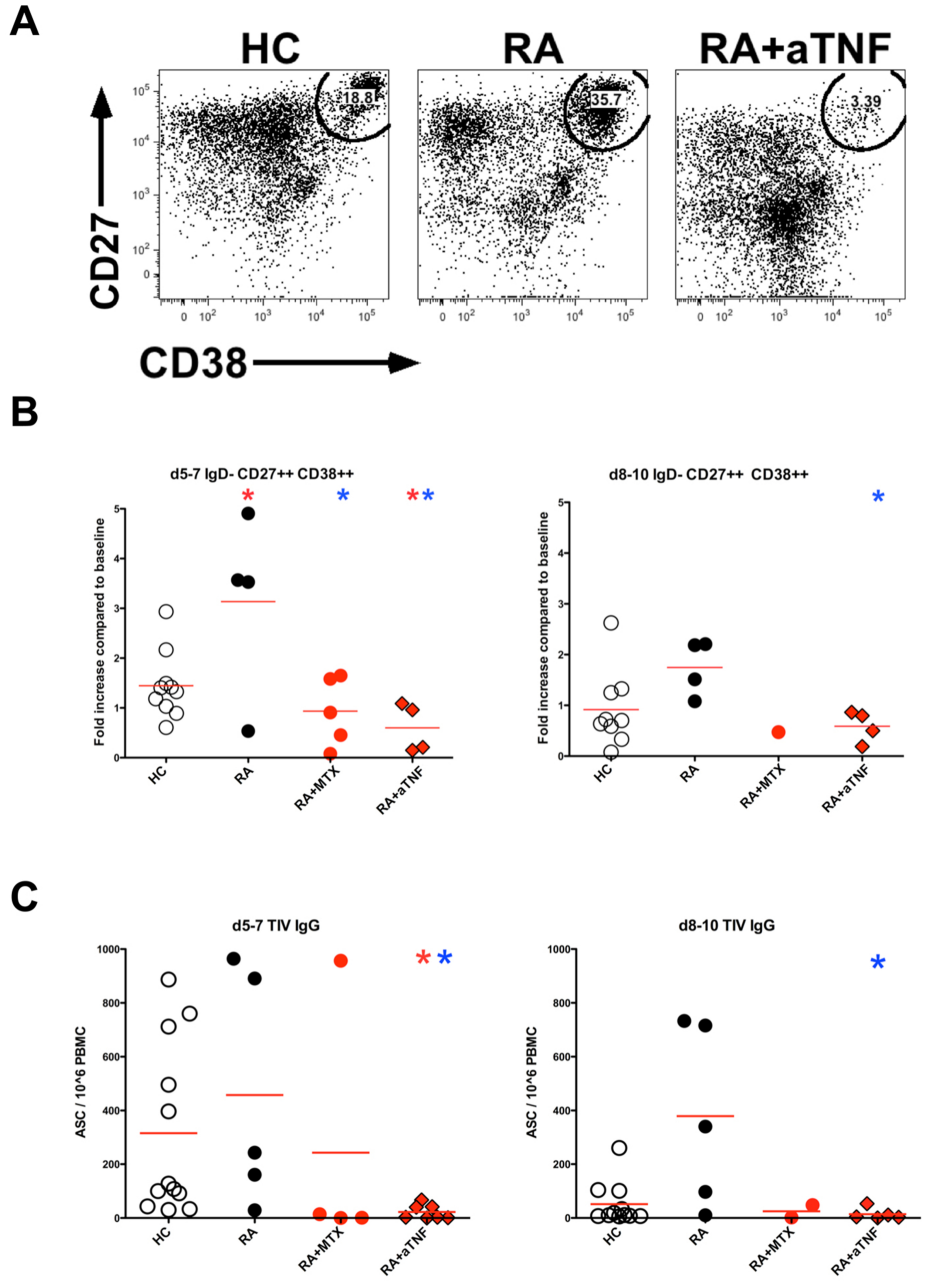
**Plasmablast and antibody secreting cell response**. The frequency of peripheral blood plasmablasts (IgD-CD24-CD27++CD38++) among total CD19+ B cells was determined by flow cytometry at baseline, day five to day seven, and day eight to day ten following vaccination with seasonal trivalent influenza vaccine (TIV). (**a**) Identification of plasmablasts from representative subjects at day five to day seven following 2010-2011 TIV. Plots are gated on live, CD3-CD19+IgD-CD24- cells. (**b**) Fold change in plasmablast frequency over baseline following 2009-2010 TIV is indicated. (**c**) TIV IgG-specific EliSpots were performed on total peripheral blood mononuclear cells (PBMC) following 2009-2010 TIV. The red star indicates significant difference (*P *< 0.05) as compared with healthy control (HC) group, the blue star indicates significant difference as compared with rheumatoid arthritis (RA) group. Each symbol represents an individual study subject, red line indicates group mean.

Untreated RA patients had a significantly greater change in the frequency of plasmablasts compared with healthy controls (Figures 3a and 3b). By contrast, RA patients treated with anti-TNF had a significantly decreased induction of plasmablasts at days five to seven compared with both HC and untreated RA patients (Figure 3b). This effect was observed in both fresh and frozen samples and also confirmed in a limited analysis of samples following 2010-2011 TIV (Figure 3a and data not shown). To precisely examine the induction of TIV-specific IgG and IgM ASC after vaccination PBMC were assayed by EliSpot. Significantly less TIV-specific IgG ASC were observed at days five to seven in RA patients treated with anti-TNF as compared with healthy controls (Figure 3c). Furthermore RA patients treated with anti-TNF had significantly decreased TIV IgG ASC at both days five to seven and days eight to ten as compared with untreated RA patients. Similar, although non-significant trends were observed for TIV-specific IgM ASC (data not shown). These results indicate the effector B cell response to influenza vaccine is impaired in RA patients treated with anti-TNF. Decreased plasmablast and ASC responses were observed in the RA+MTX group; however, likely due to limited sample number, significance was only reached when comparing days five to seven plasmablast change compared with untreated RA patients.

**Figure 4:**
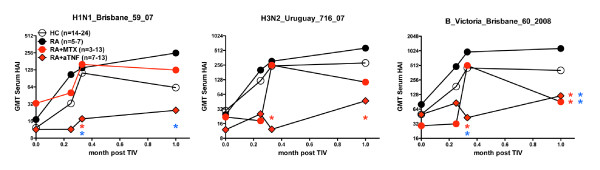
**Influenza-specific serum antibodies following 2009-2010 TIV**. Peripheral blood was obtained prior to (0) and at multiple time points within one month following vaccination with 2009 to 2010 seasonal trivalent influenza vaccine (TIV). Influenza-specific serum antibody was measured by hemagglutination inhibition assay (HAI) and geometric mean titer (GMT) determined. The red star indicates significant difference (*P *< 0.05) compared with healthy control (HC) group at same timepoint, the blue star indicates significant difference compared with the rheumatoid arthritis (RA) group at same timepoint. N, number of subjects measured for each group.

Examination of the early serum antibody response demonstrated that as soon as days eight to ten, RA patients treated with anti-TNF exhibited significantly lower HAI titers against H1, H3, and B compared with healthy controls, and significantly decreased titers to H1 and B as compared with untreated RA patients (Figure 4). At one month post-vaccination RA patients treated with anti-TNF continued to have significantly decreased HAI titers to H3 and B compared with healthy controls, and decreased HAI titers to H1, and B compared with untreated RA patients. Differences in the HAI titer of the RA+MTX as compared with HC or untreated RA group were not consistent. These results suggest RA patients treated with anti-TNF have a substantially impaired effector B cell and antibody response.

### Correlation between effector and memory responses against influenza vaccine

**Figure 5:**
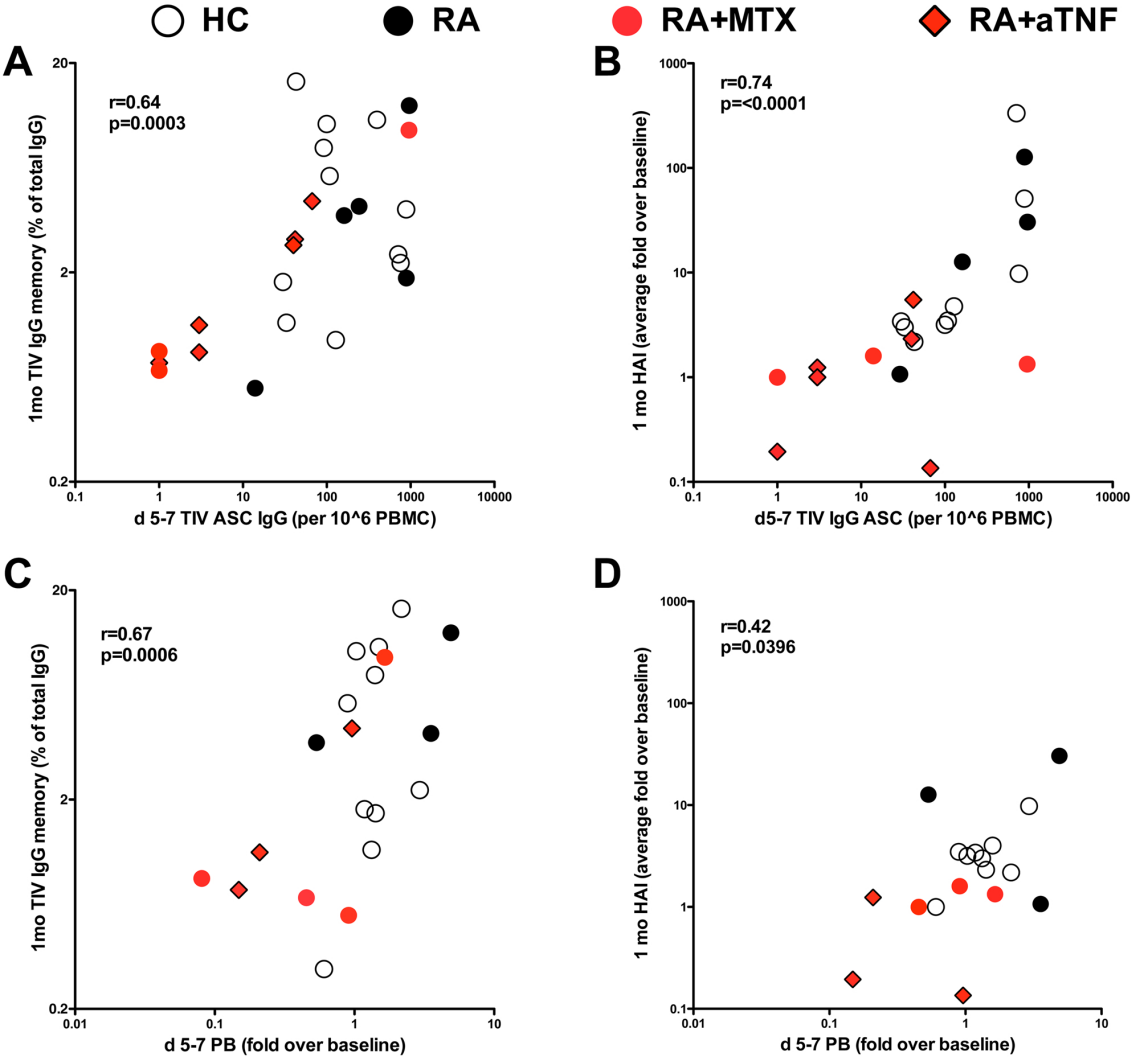
**Comparison of effector and memory response following 2009-2010 TIV**. (**a**) Trivalent influenza vaccine (TIV) IgG antibody-secreting cells (ASC) at day five to day seven vs. frequency of IgG TIV-specific memory B cells and (**b**) change in hemagglutination inhibition (HAI) titer as measured at one month. Fold change in IgD-CD27++CD38++ plasmablasts at (**c**) day five to day seven vs. frequency of IgG TIV-specific memory B cells and (**d**) change in HAI titer as measured at one month. Each symbol represents an individual study subject. Healthy controls (HC; *n *= 9 to 11), rheumatoid arthritis (RA; *n *= 3 to 5), RA+methotrexate (MTX; *n *= 3 to 4), RA+aTNF (*n *= 3 to 6). Spearman one-tailed correlation co-efficient (r) and significance (*P*) is indicated.

We previously determined that the peak of TIV-specific peripheral blood ASC shortly after vaccination correlates with the peak in HAI titer after vaccination [16]. However, it is not clear if the effector response measured by EliSpot for ASC or by flow cytometry for plasmablasts correlates with the frequency of TIV-specific memory cells and how this may be impacted in RA patients treated with anti-TNF. We thus asked if the decreased effector response to influenza vaccine in RA patients treated with anti-TNF is associated with the observed decrease in memory B cell and serum antibody on a per-patient basis. We observed a significant positive correlation of the IgG TIV-specific ASC measured at days five to seven post-vaccination with the frequency of IgG TIV-specific memory B cells measured at one month (r = 0.64, *P *= 0.0003; Figure 5a) and as expected with the change in HAI titer at one month (r = 0.74, *P *< 0.0001; Figure 5b). Additionally, the induction of CD19+IgD-CD27++CD38++ plasmablasts at days five to seven also significantly correlated with the frequency of IgG TIV-specific memory B cells (r = 0.67, *P *= 0.0006; Figure 5c) and the change in HAI titer at one month (r = 0.42, *P *= 0.0396; Figure 5d).

The subjects that had the lowest effector and memory B cell and antibody responses were primarily RA patients treated with MTX and/or anti-TNF, suggesting that these therapies directly impact the patients' acute responsiveness to TIV and subsequent generation of TIV-specific memory B cells and serum antibodies.

## Discussion

We have demonstrated that RA patients treated with anti-TNF have impaired B cell and antibody responses to seasonal influenza vaccine. This suboptimal response includes lower serum HAI antibodies, which is consistent with previous studies [4-6] and also diminished induction of influenza-specific effector and memory B cell responses, which have not been previously recognized.

The impact of anti-TNF therapy was most evident during the effector phase (day 5 to day 10) than the memory phase (one to six months) of the B cell response. This difference most likely reflects the sensitive resolution of vaccine response provided at these early time-points. Whereas resolution of the influenza-specific B cell and antibody responses at one month or later after vaccination is confounded by variation in amounts of pre-existing memory B cells and antibody present among individuals, potentially underestimating the responsiveness to a single vaccination event. The strong positive correlation observed between the effector B cell response from day five to day seven after vaccination and the memory B cell and antibody response at one month suggests a predictive value for examining the effector B cell response as an early biomarker for the development of memory B cells and antibody. The reduced effector and memory B cell response we observed in anti-TNF-treated patients, may contribute to the increased risk and severity of infection observed in anti-TNF-treated patients.

Treatment with anti-TNF is likely to target multiple aspects of the vaccine response that are contributing to the decreased B cell response observed. Previously, we demonstrated that lymphoid architecture is altered in RA patients treated with etanercept, including the destruction of the FDC network and disruption of germinal centers and corresponding decrease in peripheral blood memory B cells [13]. This dysregulation may be preventing influenza-specific B cells from participating in optimal germinal center reactions, thereby compromising the magnitude and quality of the effector and memory influenza-specific B cell response. Furthermore, as autocrine TNF enhances B cell proliferation [23], and TNF is produced in abundance by plasma cells [24]; TNF blockade may inhibit this process, contributing to the reduced development of influenza-specific memory B cells and plasmablasts that we observed. A possible additional and not mutually exclusive point of impact for TNF blockade is limiting the maintenance of influenza-specific plasma cells, because TNF enhances plasma cell survival *in vitro *[25], and has been suggested to be important for plasma cell survival *in vivo *[26,27]. It would be of particular interest to determine if TNF blockade is merely blocking the activation and expansion of influenza-specific plasmablasts and memory B cells or promoting the development of a distinct short-lived effector population that does not produce antibody, nor can develop adequately into antibody-producing cells.

Diminished B cell and antibody responses may also result from impaired monocyte, dendritic, and T cell function by TNF-blockade that has been reported, limiting the supportive, yet potentially critical roles these cells play in the induction of a robust B cell response. The known suppressive effect of anti-TNF on memory and effector T cell functions such as IFNγ and IL-2 production [28], and increased Treg function [29,30] may negatively impact the B cell response. A limitation of the current study is the inability to adequately assess the various anti-TNF agents individually for their impact on influenza vaccine response. We expect that slight differences in the B cell response to vaccination may become apparent in a large multi-anti-TNF agent comparison study; however, it is uncertain that it would be feasible to dissect differences that occur as a result of mode of action, from those that could result from the different routes of administration or dosing schedules for the various agents.

Interestingly, we observed subtle indications of enhanced B cell responsiveness to the influenza vaccine in RA patients not treated with MTX or anti-TNF as compared with the healthy control subjects. This outcome may reflect an inflammatory environment that could be present in these patients, resulting from the disease process, contributing in an adjuvant-like manner, thereby enhancing the germinal center reaction, and consequently the plasmablast and memory B cell responses. Additionally, rheumatoid factor-expressing B cells exhibit an enhanced ability to internalize antigen-immunoglobulin complexes and subsequently present the antigen to specific T cells [31], raising this possibility as a potential mechanism of enhanced vaccine responses in some RA patients. Future studies with greater numbers of untreated RA patients are warranted to adequately substantiate and dissect these questions further. Such studies may elucidate additional mechanistic aspects of the inflammatory processes in RA disease and provide strategies to enhance vaccine responsiveness in the general population.

Although a decreased response to the influenza vaccine was observed in RA patients treated with anti-TNF, it is important to emphasize that vaccination did increase serum antibody and presumed protection from infection. Furthermore, recent European League Against Rheumatism (EULAR) recommendations indicate that influenza vaccination should be strongly considered, and that vaccination can be administered during the use of anti-TNF therapy [32]. The anti-TNF treated patient population may benefit from the high-dose influenza vaccine recently licensed for individuals aged 65 years and older, and warrants further investigation. Moreover, our results indicate that every effort should be made to provide annual influenza immunization and indicated vaccination updates to RA patients before anti-TNF therapy is initiated. It is also possible that the efficacy of annual influenza vaccination in anti-TNF treated patients could be improved by timing immunization with the decline in biological effect of the corresponding agent. This question could be addressed by studying the relative efficacy of immunization provided just before the next dose of anti-TNF. The clinical value of examining anti-TNF and vaccination timing is substantiated by a recent study by Elkayam at al. which demonstrated that RA patients treated with infliximab three weeks prior to influenza vaccination had a reduced serum antibody response as compared with those patients treated with infliximab on the day of vaccination [33]. In patients with particularly high risk (i.e. those patients with previous history of severe infection, low baseline HAI titers, and/or other co-morbidities) it may be reasonable to delay the next dose of anti-TNF drug to provide a longer window of developing a protective response.

## Conclusions

RA patients treated with anti-TNF have a diminished B cell and antibody response to the influenza vaccine. This impairment is evident in the suppressed plasmablast response, and limited induction of memory B cells and serum antibody, highlighting the role of TNF in this process. The implications of this finding should be considered in the context of the increased susceptibility to infections observed in anti-TNF treated patients and suggest the need for vaccines with enhanced immunogenicity for these patient populations.

## Abbreviations

ASC: antibody-secreting cells; GMT: geometric mean titer; HAI: hemagglutination inhibition assay; HC: healthy controls; MTX: methotrexate; NF: nuclear factor; PBMC: peripheral blood mononuclear cells; PBS: phosphate buffered saline; RA: rheumatoid arthritis; TIV: trivalent influenza vaccine; TNF: tumor necrosis factor.

## Competing interests

JJK has received grant/research support from Biogen. FE-HL has received grant/research support from Trellis Biosciences, Inc. JT has received grant/research support from Protein Sciences Corp., GlaxoSmithKline, Sanofi, Pfizer, Bavarian Nordic, PaxVax, Ligocyte, and Vaxinnate and has received honoraria from ITS, Inc. and Novartis. IS has received grant/research support from Biogen and has performed consulting work for Genetech and GlaxoSmithKline.

## Authors' contributions

All authors were involved in drafting the article or revising it critically for important intellectual content, and all authors read and approved the final version to be published. JJK had full access to all of the data in the study and takes responsibility for the integrity of the data and the accuracy of the data analysis. The study was conceived and designed by JJK and IS. JJK, BZ, MB, PB, APA, DAT, RJL, RGT, JHA, AC, CTR, and JJT contributed to the acquisition of data. Data was analyzed and interpreted by JJK, AFR, JJT, CF, FE-HL and CW and IS.

## Supplementary Material

Additional file 1 **Additional Table 1. HAI seroprotection proportion**. Peripheral blood was obtained prior to (baseline), and one and six months following vaccination with trivalent influenza vaccine (TIV) throughout multiple years. Influenza-specific serum antibody was measured by hemagglutination inhibition assay (HAI) for the indicated influenza type. The number of subjects with HAI titer of 40 or higher/total number of subjects (%) are indicated. Relative difference in geometric mean titer (GMT) among rheumatoid arthritis (RA) groups as compared with the healthy control (HC) group at same time point indicated by color. Cells with bold outline indicate significant difference (*P *< 0.05) as compare with the HC group at same timepoint. Click here for file

Additional file 2 **Additional Figure 1. TIV-specific IgG memory B cell frequency**. Total B cells were isolated at baseline and one and six months following immunization with 2006/2007 through 2009/2010 trivalent influenza vaccine (TIV), cultured with CpG and IL-2 for four days, and TIV and total IgG specific EliSpots performed to determine the frequency of TIV specific memory B cells. An individual subject may have provided data from multiple study years. No statistical difference (*P *< 0.05) was detected among groups at individual time points. Click here for file
