# Supplementary material for: Decreased influenza-specific B cell responses in rheumatoid arthritis patients treated with anti-tumor necrosis factor
Source: Arthritis Res Ther. 2011 Dec 16;13(6):R209. doi: 10.1186/ar3542 (PMC3334662; doi:10.1186/ar3542)
Supplement: Additional file 1 — Additional Table 1. HAI seroprotection proportion. Peripheral blood was obtained prior to (baseline), and one and six months following vaccination with trivalent influenza vaccine (TIV) throughout multiple years. Influenza-specific serum antibody was measured by hemagglutination inhibition assay (HAI) for the indicated influenza type. The number of subjects with HAI titer of 40 or higher/total number of subjects (%) are indicated. Relative difference in geometric mean titer (GMT) among rheumatoid arthritis (RA) groups as compared with the healthy control (HC) group at same time point indicated by color. Cells with bold outline indicate significant difference (P < 0.05) as compare with the HC group at same timepoint. [file ar3542-S1.PDF]

		H1			H3			B		
		baseline	1 mo	6 mo	baseline	1 mo	6 mo	baseline	1 mo	6 mo
06-07	HC	41/48 (85)	46/47 (98)	40/43 (93)	24/48 (50)	47/47 (100)	40/43 (93)	33/48 (69)	46/47 (98)	39/43 (91)
	RA+aTNF	12/15 (80)	13/14 (93)	12/13 (92)	8/15 (53)	13/14 (93)	11/13 (85)	11/15 (73)	13/14 (93)	12/13 (92)
07-08	HC	41/58 (71)	54/54 (100)	44/44 (100)	54/58 (93)	54/54 (100)	43/44 (98)	50/58 (86)	54/54 (100)	43/44 (98)
	RA	5/12 (42)	10/12 (83)	9/12 (75)	7/12 (58)	10/12 (83)	10/12 (83)	12/12 (100)	12/12 (100)	11/12 (92)
	RA+MTX	20/41 (49)	28/32 (88)	21/25 (84)	32/41 (78)	30/32 (94)	23/25 (92)	33/41 (80)	31/32 (97)	24/25 (96)
	RA+aTNF	21/30 (70)	35/36 (97)	25/29 (86)	25/30 (83)	34/36 (94)	29/29 (100)	25/30 (83)	35 /36 (97)	27/29 (93)
08-09	HC	11/25 (44)	17/22 (77)	15/23 (65)	13/25 (52)	21/22 (95)	21/23 (91)	10/25 (40)	17/22 (77)	16/23 (70)
	RA	10/18 (56)	15/18 (83)	13/17 (76)	11/18 (61)	16/18 (89)	15/17 (88)	13/18 (72)	17/18 (94)	16/17 (94)
	RA+MTX	13/25 (52)	22/24 (92)	17/20 (85)	16/25 (64)	22/24 (92)	16/20 (80)	16/25 (64)	18/24 (75)	16/20 (80)
	RA+aTNF	10/23 (43)	16/22 (73)	9/17 (53)	12/23 (52)	16/22 (73)	7/17 (41)	12/23 (52)	15/22 (68)	9/17 (53)
09-10	HC	10/23 (43)	19/23 (83)	12/23 (52)	12/23 (52)	22/23 (96)	12/23 (52)	16/23 (70)	22/23 (96)	16/23 (70)
	RA	3/7 (43)	6/6 (100)	2/3 (67)	4/7 (57)	6/6 (100)	3/3 (100)	6/7 (86)	6/6 (100)	2/3 (67)
	RA+MTX	9/13 (69)	11/12 (92)	9/12 (75)	9/13 (69)	11/12 (92)	9/12 (75)	8/13 (62)	10/12 (83)	7/12 (58)
	RA+aTNF	4/13 (31)	7/13 (54)	7/14 (50)	4/13 (31)	8/13 (62)	8/14 (57)	7/13 (54)	11/13 (85)	5/14 (36)

Change as compared to HC

- > 180%
- 160 – 179%
- 140 – 159%
- 120 – 139%
- 81 – 119%
- 61 – 80%
- 41 – 60%
- 21 – 40%
- < 21%

Additional Table 1. HAI Seroprotection Proportion. Peripheral blood was obtained prior to (baseline), 1 and 6 months following vaccination with TIV throughout multiple years. Influenza-specific serum antibody was measured by hemagglutination inhibition assay for the indicated influenza subtype. The number of subjects with HAI titer ≥ 40 / total number of subjects (%) are indicated. Relative difference in GMT among RA groups as compared with HC group at same timepoint indicated by color. Cells with bold outlines indicate significant difference (p<0.05) as compare with HC group at same timepoint.
