# Supplementary material for: Decreased influenza-specific B cell responses in rheumatoid arthritis patients treated with anti-tumor necrosis factor
Source: Arthritis Res Ther. 2011 Dec 16;13(6):R209. doi: 10.1186/ar3542 (PMC3334662; doi:10.1186/ar3542)
Supplement: Additional file 2 — Additional Figure 1. TIV-specific IgG memory B cell frequency. Total B cells were isolated at baseline and one and six months following immunization with 2006/2007 through 2009/2010 trivalent influenza vaccine (TIV), cultured with CpG and IL-2 for four days, and TIV and total IgG specific EliSpots performed to determine the frequency of TIV specific memory B cells. An individual subject may have provided data from multiple study years. No statistical difference (P < 0.05) was detected among groups at individual time points. [file ar3542-S2.PDF]

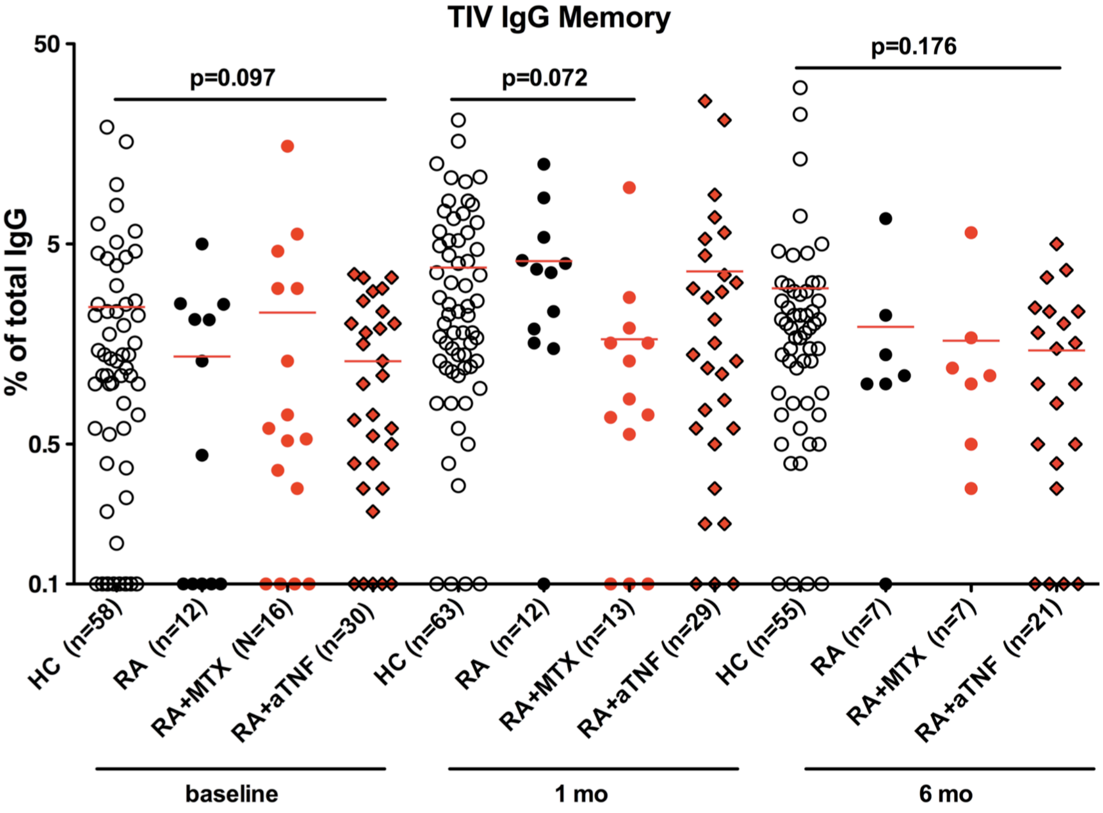

Additional Figure 1. TIV-specific IgG memory B cell frequency. Total B cells were isolated at baseline and 1 and 6 months following immunization with 2006/2007 through 2009/2010 TIV, cultured with CpG and IL-2 for 4 days, and TIV and total IgG specific EliSpots performed to determine the frequency of TIV-specific memory B cells. An individual subject may have provided data from multiple study years. No statistical difference ($p<0.05$) was detected among groups at individual time points.
